# Study on the Promotion of Bacterial Biofilm Formation by a *Salmonella* Conjugative Plasmid and the Underlying Mechanism

**DOI:** 10.1371/journal.pone.0109808

**Published:** 2014-10-09

**Authors:** Zhen Liu, Fengxia Que, Li Liao, Min Zhou, Lixiang You, Qing Zhao, Yuanyuan Li, Hua Niu, Shuyan Wu, Rui Huang

**Affiliations:** Medical College of Soochow University, Suzhou, P. R. China; The Biodesign Institute, Arizona State University, United States of America

## Abstract

To investigate the effect of the pR_ST98_ plasmid, originally isolated from *Salmonella enterica* serovar Typhi (*S. Typhi*), on biofilm (BF) formation, we carried out *in vitro* experiments using *S. Typhi*, *Salmonella enterica* serovar Typhimurium (*S. Typhimurium*) and *Escherichia coli* (*E. coli*). We further explored the effects of pR_ST98_ *in vivo* by establishing two animal models, a tumor-bearing mouse model and a mouse urethral catheter model. Moreover, we examined the relationship between the quorum-sensing (QS) system and pR_ST98_-mediated BF formation. These studies showed that pR_ST98_ enhanced BF formation in different bacteria *in vitro*. In both animal models, pR_ST98_ promoted BF formation and caused more severe pathological changes. It was previously reported that *Salmonella* senses exogenous N-acylhomoserine lactones (AHLs) through the regulatory protein SdiA and regulates the expression of genes including the virulence gene *rck*, which is located on the virulence plasmid of some serotypes of *Salmonella*. In this study, we confirmed the locus of the *rck* gene on pR_ST98_ and found that AHLs increased *rck* expression in pR_ST98_-carrying strains, thereby enhancing bacterial adherence, serum resistance and bacterial BF formation. In conclusion, the *Salmonella* conjugative plasmid pR_ST98_ promotes bacterial BF formation both *in vitro* and *in vivo*, and the mechanism may relate to the AHL-SdiA-Rck signaling pathway.

## Introduction

*Salmonella*, a facultative anaerobic bacterium that has a broad range of hosts including humans, farm animals and plants, causes serious infection and thousands of deaths each year, posing a significant threat to humans.

**Figure 1:**
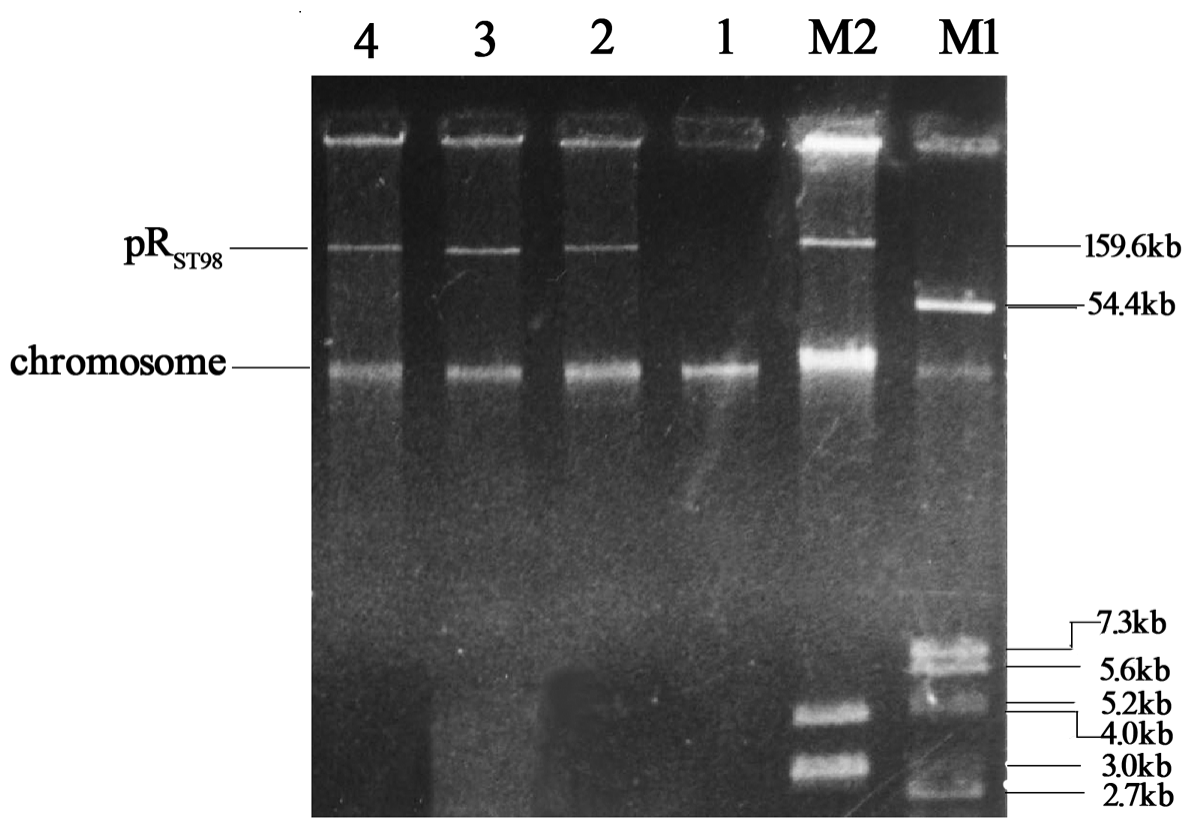
Electrophoresis plasmid profile of pR_ST98_. Lane M1, *S. flexneri_24570_*, plasmid size marker; Lane M2, *E. coli* V_517_, plasmid size marker; Lane 1–3, multi-drug resistant *S. Typhi* used as representative strains that naturally harbored pR_ST98_ and were resistant to chloramp henicol, streptomycin, trimethoprim and sulphonamide, gentamicin, neomycin, kanamycin, cephalosporin ampicillin, carbenicillin and tetracycline; Lane 4, antibiotic-sensitive *S. Typhi*, which were plasmid free, and used as the negative control.

A large outbreak of *Salmonella enterica* serovar Typhi (*S. Typhi*) infection occurred in the 1980s. Five hundred ninety-one strains were isolated from the blood of patients who had acute and severe clinical symptoms. It was shown that more than 80% of isolates were multi-drug resistant, which was attributed to a large plasmid (R plasmid) with a size of 159 kb, designated as pR_ST98_, belonging to the IncC group (**Fig. 1**) [1]. Our previous study showed that pR_ST98_ is a chimerical plasmid carrying genes responsible for drug resistance and virulence. The strains harboring pR_ST98_ were found resistant to trimethoprim, streptomycin, kanamycin, sulfonamide, neomycin, gentamicin, chloramphenicol, tetracycline, carbenicillin, ampicillin, and cephalosporin. It was confirmed in our previous studies that pR_ST98_ contains a DNA sequence homologous to the *Salmonella* plasmid virulence gene (*spv*), which was found in all pathogenic *Salmonella* spp. except *S. Typhi*. The sequence of the ORF (open reading frame) of *spvR* and *spvB* on pR_ST98_ shared more than 99% similarity with that of *spvR* and *spvB* on the virulence plasmid in *Salmonella enterica* serovar Typhimurium (*S. Typhimurium*) [2], indicating the presence and distribution of *spv* in *Salmonella*. Later studies demonstrated that pR_ST98_ increased the serum resistance of *Salmonella*, promoted *S. Typhi* survival in macrophages *in vitro* and decreased the LD_50_ (50% lethal dose) values of *S. Typhimurium* in infected mice [3]. Recent studies in our laboratory found that pR_ST98_ had inhibitory effects on autophagy in macrophages, thus weakening the innate immunity of host cells [4]–[5]. In addition, pR_ST98_ is a conjugative plasmid that spreads easily among *S. Typhi*, *S. Typhimurium*, *Escherichia coli* (*E. coli*) and *Shigella flexneri* (*S. flexneri*) *in vitro*, and it was very easily transferred from *S. Typhimurium* to *E. coli* in mice [6]. Given these characteristics of pR_ST98_, it is expected that this plasmid plays important roles in bacterial resistance against hostile immune factors and in causing aggravated infection.

Due to their significance in the food industry and in public health, bacterial biofilms (BFs) have become the focus of studies since their first description in 1978. A biofilm is a structured community of bacterial cells enclosed in a self-produced polymeric matrix adherent to abiotic or living surfaces. Bacterial BF formation is described in three phases: initial attachment, proliferation and maturation, and detachment [7]. It was reported that approximately 80% of bacterial infections are related to BFs [8]. In the transition to BF status, some characteristics of bacteria change, including their adherence, invasion, virulence, and resistance. Therefore, it is extremely difficult to eradicate BF-related contamination using routine methods such as disinfectants [9]–[10]. Taking *Salmonella* BF as an example, Barker and Bloomfield found even when treated with cleaning products, *Salmonella* BF that developed in toilets could live up to four weeks after patients were cured of salmonellosis infections [11]. Bacterial BF formation during food processing has caused severe consequences in public health. The resistance against multiple antibiotics is greatly increased when *Salmonella* is enclosed in a BF [12], which makes BF-related diseases more difficult to treat or cure. The persistence of bacterial BFs on the surface of teeth damages the tooth enamel and induces an inflammatory reaction in the surrounding gums [13]. *S. Typhi* BFs formed on the gallbladder were reported to be associated with the occurrence of liver cancer [14]. In addition, bacterial BFs in medical implants such as indwelling catheters could led to severe consequences. Therefore, the effects of BFs on causing endocarditis and intra-abdominal, pelvic, and urinary tract infections (UTIs) have been extensively studied [12].

It has been suggested that a conjugative plasmid could promote BF formation in *E. coli* and other bacteria. This phenomenon could be attributed to conjugative-plasmid related factors. It has been proposed that the conjugative pili act as adhesion factors at the early stage of BF formation [15]. Colanic acid, curli, and adhesion dynamics in *E. coli* all contribute to conjugative plasmid-mediated BF formation [16]–[17]. Alvise *et al.* suggested that extracellular DNA (eDNA) is responsible for increased BF formation mediated by the conjugative plasmid TOL in *Pseudomonas putida* KT2440 [18]. Furthermore, type 3 fimbriae, encoded by the conjugative plasmid pOLA52, were implicated in conjugative plasmid-enhanced BF production in *E. Coli* [19]–[20]. However, very few factors conducted by conjugative plasmid were reported in *Salmonella*. Because the pR_ST98_ plasmid has the ability to conjugate, we explored the relationship between pR_ST98_ and BF formation in different *Salmonella* and *E. coli* strains by multiple methods *in vitro*, including violet dye staining, scanning electron microscopy (SEM) and confocal laser scanning microscopy (CLSM). Furthermore, two animal models were established to investigate the effects of pR_ST98_ on BF formation *in vivo*. One was a tumor-bearing mouse intravenously infected by *S. Typhimurium* χ3337*lux* and χ3337*lux*/pR_ST98_ (by the conjugal transfer of pR_ST98_ to χ3337*lux*) [21]. Here *S. Typhimurium* was used as a surrogate for *S. Typhi* because *S. Typhi* only causes human infections, and no suitable model has been established for investigation of *S. Typhi* pathogenesis. *S. Typhimurium* is a facultative anaerobic bacterium that can survive both in tumor active areas and necrosis areas. In addition, *S. Typhimurium* is driven toward tumors through chemoattraction in infections. Three important receptors, the aspartate receptor, the serine receptor, and the ribose/galactose receptor, bind to compounds released by tumor and specifically attract *S. Typhimurium* to preferentially migrate to the tumor [22]. The other animal model was a mouse with a urethral catheter infected by *E. coli* K_12_W_1485_ and *E. coli* K_12_W_1485_/pR_ST98_ (by the conjugal transfer of pR_ST98_ to *E. coli* K_12_W_1485_) because *E. coli* is one of the most common microbes in nosocomial infections.

N-acylhomoserine lactones (AHLs) are signaling molecules of the quorum sensing (QS) system, which responds to bacterial population density and triggers some gene expressions. AHLs play an important role in BF formation. Though *Salmonella* does not produce AHLs, it synthesizes the signal molecule receptor SdiA, which responds to AHLs released by other bacteria [12]. Lee found that SdiA binds extracellular signals and affects BF formation in *E. coli*; however, no direct link has been found between AHLs and BF formation in *Salmonella* [23]. Encoding an outer membrane protein, the *rck* gene on the virulence plasmid of some serotypes of *Salmonella* was regulated by SdiA. It was found that the *rck* operon affects the expression of plasmid-encoded fimbriae, which were shown to be vital components of the extracellular matrix and to promote BF formation [24]–[25]. In this study, we investigate the effects of pR_ST98_ on BF formation and its interactions with the AHLs-SdiA-Rck pathway.

## Materials and Methods

### Bacteria and culture conditions

The bacteria used in our study were listed in **Table 1**. Bioluminescent strains of *S. Typhi* and *S. Typhimurium* were constructed by electroporation of the pBEN276 plasmid containing a constitutive *lux* expression cassette, and the *lux* expression cassette recombined within the bacterial chromosome according to reference [26]. The use of bioluminescent bacteria provides an effective tool in the detection of *S. Typhimurium* BF formation *in vivo*. These strains were grown to mid-logarithmic phase in Luria-Bertani (LB) medium at 37°C, with a shaking speed of 200 r.p.m. Ampicillin was added into the medium at a concentration of 100 µg/ml to maintain the stability of the pR_ST98_ plasmid in some strains. The bacterial population density was determined by measuring *OD_600_* values with a spectrophotometer.

**Table 1:** Strains used in this study.

Background	Strains	Relevant characteristics	Reference
*Salmonella Typhi* (*S. Typhi*)	ST_8_	wildtype with the resistent plasmid pR_ST98_ Tc^r^ Amp^r^ Cm^r^ Sm^r^ Kn^r^ Cb^r^ Gm^r^ Nm^r^ Su^r^ Tmp^r^ Cp^r^	5
	ST_8_-ΔpR_ST98_	deletion of plasmid pR_ST98_ from ST_8_ Tc^s^ Amp^s^ Cm^s^ Sm^s^ Kn^s^ Cb^s^ Gm^s^ Nm^s^ Su^s^ Tmp^s^ Cp^s^	5
	ST_8_-c-pR_ST98_	conjugal transference of pR_ST98_ to ST_8_-ΔpR_ST98_ Tc^r^ Amp^r^ Cm^r^ Sm^r^ Kn^r^ Cb^r^ Gm^r^ Nm^r^ Su^r^ Tmp^r^ Cp^r^	5
*Salmonella Typhimurium* (*S. Typhimurium*)	?3306	wildtype, pStSR100^+^, Nal^r^	8
	?3337	virulence plasmid-cured derivative of χ3306, Spv, Nal^r^	8
	?3337/pR_ST98_	conjugal transference of pR_ST98_ to χ3337, Spv, Nal^r^ Tc^r^ Amp^r^ Cm^r^ Sm^r^ Kn^r^ Cb^r^ Gm^r^ Nm^r^ Su^r^ Tmp^r^ Cp^r^	5
	?3306*lux*	electrotransformation of *lux* gene into χ3306 virulence plasmid positive, Nal^r^	26, in this study
	?3337*lux*	electrotransformation of *lux* gene into χ3337 virulence plasmid-cured derivative of χ3306, Spv, Nal^r^	26, in this study
	?3337*lux*/pR_ST98_	electrotransformation of *lux* gene into χ3337 conjugal transference of pR_ST98_ to χ3337*lux*, Spv, Nal^r^ Tc^r^ Amp^r^ Cm^r^ Sm^r^ Kn^r^ Cb^r^ Gm^r^ Nm^r^ Su^r^ Tmp^r^ Cp^r^	26, in this study
*Escherichia coli* (*E. coli*)	K_12_W_1485_	Rifr F^−^ Lac^+^	5
	K_12_W_1485_/pR_ST98_	conjugal transference of pR_ST98_ to K_12_W_1485_, Rif^r^ F^−^ Lac^+^ Tc^r^ Amp^r^ Cm^r^ Sm^r^ Kn^r^ Cb^r^ Gm^r^ Nm^r^ Su^r^ Tmp^r^ Cp^r^	5

### Cell lines and animals

CT26 colon carcinoma cells (ATCC CRL-2638) and HeLa cells purchased from the Cell Resource Center of Shanghai Institutes for Biological Sciences of Chinese Academy were cultured as a monolayer in RPMI1640 Medium (Sigma, America) supplemented with 10% (v/v) heat-inactivated fetal calf serum (Thermo Scientific, America). Six- to seven-week-old female BALB/c mice were purchased from the Experimental Animal Center of Soochow University.

### Ethics statement

All animal experiments were approved by the Animal Experimental Committee of the Soochow University (Grant 2111270) and were in accordance with the National Institutes of Health Guidelines for the Care and Use of Laboratory Animals (NIH Guidelines).

### Comparison of BF by crystal violet staining

Bacteria cultured overnight in LB medium were diluted to *OD_600_* 0.4. BF formation in polystyrene microtiter plates was assayed as described by O′Toole & Kolter [27] with modification. Briefly, cells were grown in the wells of the microtiter plates in 200 µl of LB medium supplemented with 1% glucose for 72 h at 30°C. The medium was then removed and replaced by 200 µl of a 1% (w/v) solution of crystal violet. After incubation at room temperature for 15 min, the dye was removed, and the wells were washed thoroughly with phosphate buffered saline (PBS). Following drying, BFs were observed with inverted microscopy and imaged. To quantify the attached bacteria, the crystal violet was solubilized with 200 µl of 30% (v/v) acetic acid solution, and the absorbance was measured at 570 nm (i.e., *OD*_570_) in an ELISA reader (Biotek). The experiment was repeated three times with each sample in 4 wells.

### Observation of BF structure with CLSM

Bacteria were cultured in the 24-well polystyrene plates at 30°C for 72 h. The pellicles collected from the air-broth interface were placed on the microscope slides and stained with 0.01% Acridine Orange (AO). After sealed with 40% glycerine, the samples were observed with a Leica TCS-SP2 CLSM. Imaging was performed using the 40*/1.3 objective, and simulated three-dimensional images were generated with COMATAT software. The experiment was repeated three times with duplicate samples.

### Detection of BF using SEM

The cultured pellicles were transferred to cover slips pre-coated with lysine, followed by fixation with 4% glutaraldehyde and post-fixation with 1% osmic acid before dehydration with a graded series of tert-butyl alcohol dilutions (30 to 100%). After the critical point in drying, the samples were observed with an xL-20 scanning electron microscope (Philip, Netherlands).

### BF formation in two different animal models *in vivo*

For the tumor-bearing BALB/c mouse model, each group of six was subcutaneously inoculated with 1×10^6^ CT26 cells at the pre-abdomen site. When the tumor reached a diameter of 5–8 mm, the tumor-bearing mice were injected intravenously with 1×10^7^ CFU of *S. Typhimurium* χ3337*lux* or χ3337*lux*/pR_ST98_ in PBS. In-vivo imaging was performed at 1 d, 2 d and 3 d post-infection (p.i.) using an FX Pro in-vivo imaging system (IVIS, DXS4000pro) to observe the injected bacteria in mice. Mice were sacrificed at 3 d p.i., and tumors, livers, and spleens were collected for SEM and colony forming unit (CFU) analysis.

For the urethral catheter model, polyethylene tubes (PE10 with inside and outside diameter of 0.28 mm and 0.6 mm, respectively) pretreated with 75% ethanol and UV sterilized for 12 h, were incubated with *E. coli* K_12_W_1485_ or with *E. coli* K_12_W_1485_/pR_ST98_ for 1 d. Female mice in each group of six were anesthetized by injecting 10% chloral hydrate in the enterocoelia. The periurethral area was sterilized with 75% ethanol, and the pre-incubated PE10 tubes were gently inserted transurethrally. PE10 tubes, livers and kidneys were aseptically collected from sacrificed mice on 5 d and 8 d p.i., and washed with PBS. PE10 tubes were fixed in glutaraldehyde for SEM or stained with 0.01% AO staining solution for CLSM. In addition, PE10 tubes, as well as livers and kidneys, were sonicated for 20 min in PBS for CFU counting. For the preparation of paraffin sections, livers and kidneys fixed in 10% (v/v) paraformaldehyde were embedded in paraffin wax, sectioned with a thickness of 3–4 µm, placed on slides and stained with hematoxylin-eosin (H&E) staining solution.

### Analysis of the mechanism of pR_ST98_ promoted BF formation by adherence assay

HeLa cells were seeded in 24-well tissue culture plates at 10^5^ cells per well and incubated at 37°C and 5% CO_2_ for 12 h. Cells were infected with ST8, ST_8_-c-pR_ST98_ or ST_8_-ΔpR_ST98_ with an MOI of 100∶1 in the presence of 1 µM C8-AHLs dissolved by DMSO (Sigma, America) or saline. The plates were incubated at 37°C with 5% CO_2_ for 60 min, and the cells were washed three times with PBS before lysing with 200 µl 0.2% Triton X-100 for 30 min at 37°C. The supernatant was collected for CFU counting. Each bacterial strain was assayed in triplicate, and experiments were repeated twice.

### Serum resistance

Serum collected from 5 healthy rabbits and guinea pigs was filter-sterilized. *S. Typhi* were cultured in LB for 16 h at 37°C, gradual diluted *OD_600_* value to 1×10^4^ CFU/ml. Then, 20 µl bacterial cultures were incubated with 200 µl serum plus 1 µM C8-AHLs or saline 2 h at 37°C. CFUs were enumerated to count the surviving bacteria. The experiment was repeated twice with triplicate samples.

### PCR and sequencing of *rck* gene

Genomic DNA was extracted from ST8, ST_8_-c-pR_ST98_ and ST_8_-ΔpR_ST98_ by boiling. PCR was performed using primers *rck*-F: 5′-GTTGTATCCCGGCATGCTGA-3′ and *rck*-R: 5′-ATATTGCCCAGAGCCGGATAGAG-3′ [28]. to detect the *rck* gene located on pR_ST98_. Then, the gene was linked to the pEJT1.2 plasmid and transduced into *E. coli* TOP10. The *rck* gene was sequenced.

### RT-PCR of *rck* gene

Total RNA extraction was performed using the Total RNA kit I (OMEGA bio-tek, America). The samples were centrifuged at 4000 r.p.m. for 10 min, and the supernatant was discarded. The pellet was resuspended in 100 µl lysis buffer (50 mg/ml lysozyme in Tris-EDTA buffer) and incubated at room temperature for 7 min. The subsequent steps of the RNA purification were performed according to the manufacturers' instructions. The quality of the isolated RNA was assessed via gel electrophoresis (PowerPac Basic, America). RNA concentrations were determined using the NanoDrop System (Thermo Scientific, America). The expression of the *rck* gene was determined by SuperScript TM III platinum One-Step Quantitative RT-PCR System (Invitrogen, America) according to the manufacturers' instructions. The reaction solution contained 25 µl of 2× reaction mixes, 1 µl of TaqMix, 0.2 µl of specific primers, 2 µl of mRNA, and 21.6 µl of DEPC water. Reactions were performed on a PCR system (MJ Research, America). cDNA was first produced in the RT step with 50°C for 15 min, followed by a DNA amplification step at 95°C, 5 min for denaturing, and 35 cycles (95°C for 40 s, 55°C for 30 s and 72°C for 115 s). The DNA product was observed and analyzed by gel electrophoresis and an automatic gel imaging analysis system (Syngene, UK). The primers used in this experiment were *rck*-F and *rck*-R.

### C8-AHLs on BF formation

ST_8_ *lux*, ST_8_-ΔpR_ST98_ *lux* and ST_8_-c-pR_ST98_ *lux* were cultured in the 24-well polystyrene plates at 30°C for 24 h adding 1µM C8-AHLs in the experimental group and 1µM saline in the control group. The media were then removed and washed thoroughly with PBS for 3 times. BFs were observed with IVIS.

### Statistical methods

Data among groups were compared by three independent analyses, using an unpaired two-tailed Student *t* test, a one-way ANOVA, and a SNK-q (Student-Newman-Keuls) analysis. Among all the analyses, a *p* value <0.05 was considered statistically significant. All the experiments were repeated three times with duplicate samples.

## Results

### 1. The promotion effects of pR_ST98_ on BF formation in different bacteria *in vitro*

**Figure 2:**
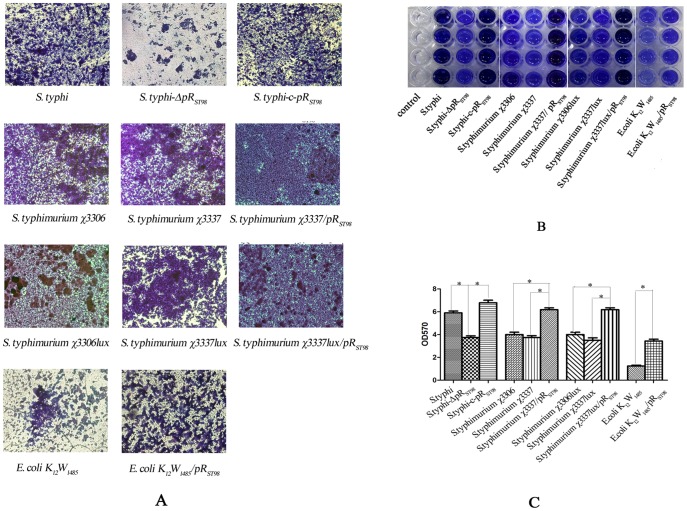
Comparison of BF developed by different bacteria. (**A**) Different bacteria cultured *in vitro* for 3 d in microtiter plates at 30°C and stained by crystal violet (400×). (**B**) Different bacteria cultured *in vitro* for 3 d in 96-well plates at 30°C and stained by crystal violet. (**C**) Optical density of cultures measured at a wavelength of 570 nm (*OD_570_*) after crystal violet staining (*p <0.05).

To study the effect of the plasmid pR_ST98_ on BF formation in different strains, several methods were employed, including crystal violet staining, CLSM, and SEM. Including *S. Typhi* ST_8_, *S. Typhimurium* χ3306 (the bioluminescent *S. Typhimurium* strains were also studied), *E. coli* K_12_W_1485_ and their derivatives, three groups of bacteria were used in the crystal violet staining method to compare their ability to form BFs. For the intra-group comparison in the ST_8_ group, ST_8_ and ST_8_-c-pR_ST98_ were found to develop thicker BFs than ST_8_-ΔpR_ST98_ (*p* <0.05) (**Fig. 2A**). Consistently, BFs formed by *S. Typhimurium* carrying pR_ST98_ were significantly more robust compared with those without pR_ST98_ in the χ3306*lux* group, which included χ3306*lux*, χ3337*lux* and χ3337*lux*/pR_ST98_ strains (*p* <0.05) (**Fig. 2A**). Similarly, *E. coli* K_12_W_1485_/pR_ST98_ had a stronger ability to form BFs than *E. coli* K_12_W_1485_ (*p* <0.05). These results indicate that pR_ST98_ plays an important role in promoting BF formation. For the inter-group comparison, *Salmonella* developed thicker BFs than *E. coli* did, and the difference was even more significant when both *Salmonella* and *E. coli* harbored pR_ST98_, suggesting that pR_ST98_ might enhance BF formation in *Salmonella* more strongly than in *E. coli*. Meanwhile, the *lux* gene was shown to have no effect on BF formation (data not shown), and there was no difference observed between χ3306 and χ3337 (**Fig. 2A to C**).

**Figure 3:**
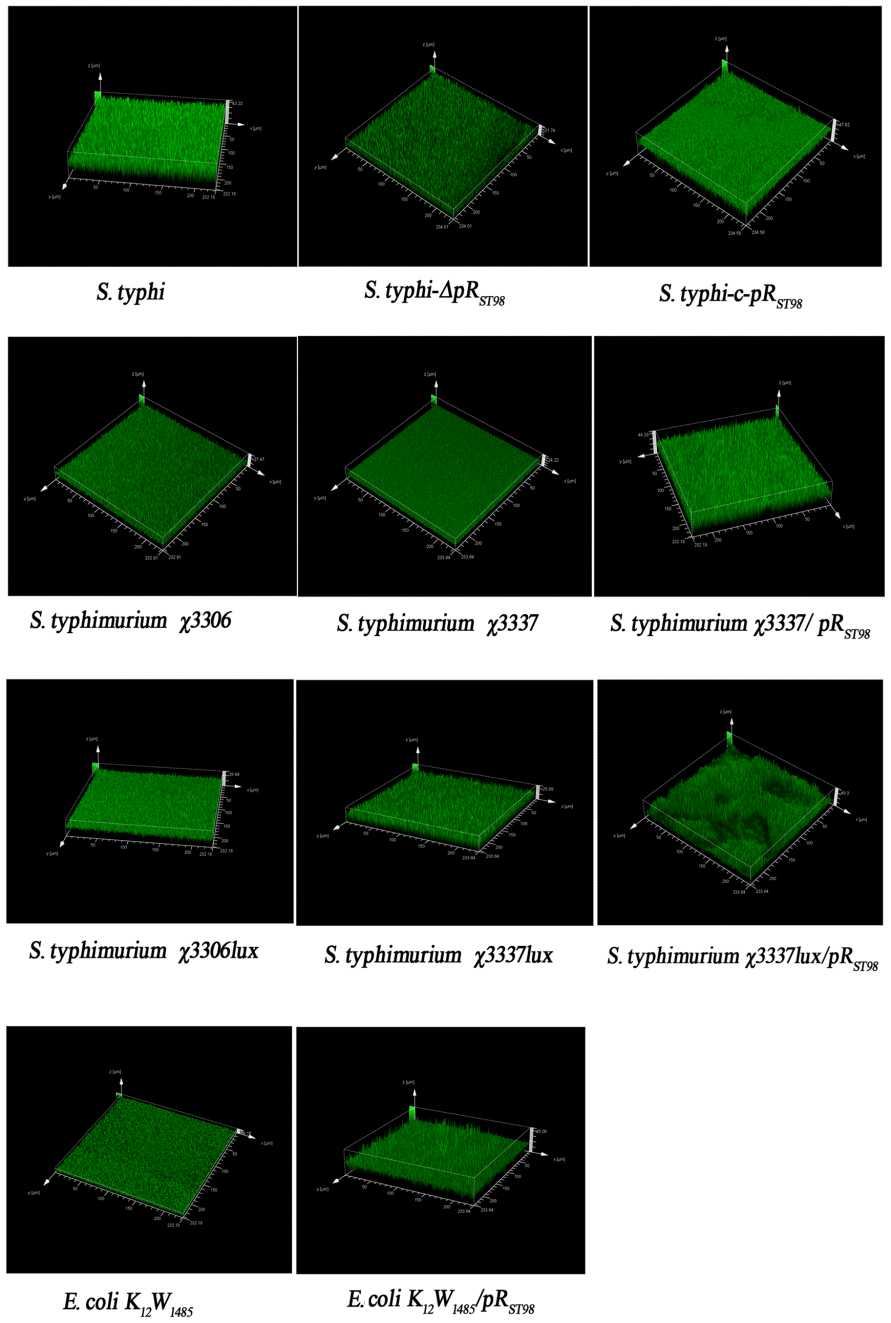
Quantification of BF by CLSM. Different bacteria were cultured in 24-well plates for 3 d, and the developed pellicles were harvested, placed on glass slides, and subjected to 3D image reconstruction by CLSM.

Bacteria harboring pR_ST98_ developed slimy and viscous pellicles, while pR_ST98_-free bacteria formed loose and less coherent BFs [13]. Tomography and three-dimensional reconstruction by CLSM showed that BFs in *S. typhi* ST_8_ and ST_8_-c-pR_ST98_ were developed with 43.23 µm and 47.62 µm thicknesses, respectively, which were much thicker than that in ST_8_-ΔpR_ST98_ with a thickness of 21.74 µm; *S. Typhimurium* harboring pR_ST98_ was a stronger BF developer (χ3337: 24.22 µm vs χ3337/pR_ST98_: 44.33 µm; χ3337*lux*: 25.89 µm vs χ3337*lux*/pR_ST98_: 40.30 µm); *E. coli* K_12_W_1485_ produced a BF of 9.1 µm in thickness, while the BF developed by *E. coli* K_12_W_1485_/pR_ST98_ had a thickness of 45.06 µm. The BF thicknesses of *S. Typhimurium* χ3306 and χ3306*lux* were 28.23 and 27.98 µm, which were not significantly different from *S. Typhimurium* χ3337 and χ3337*lux* (**Fig. 3**).

**Figure 4:**
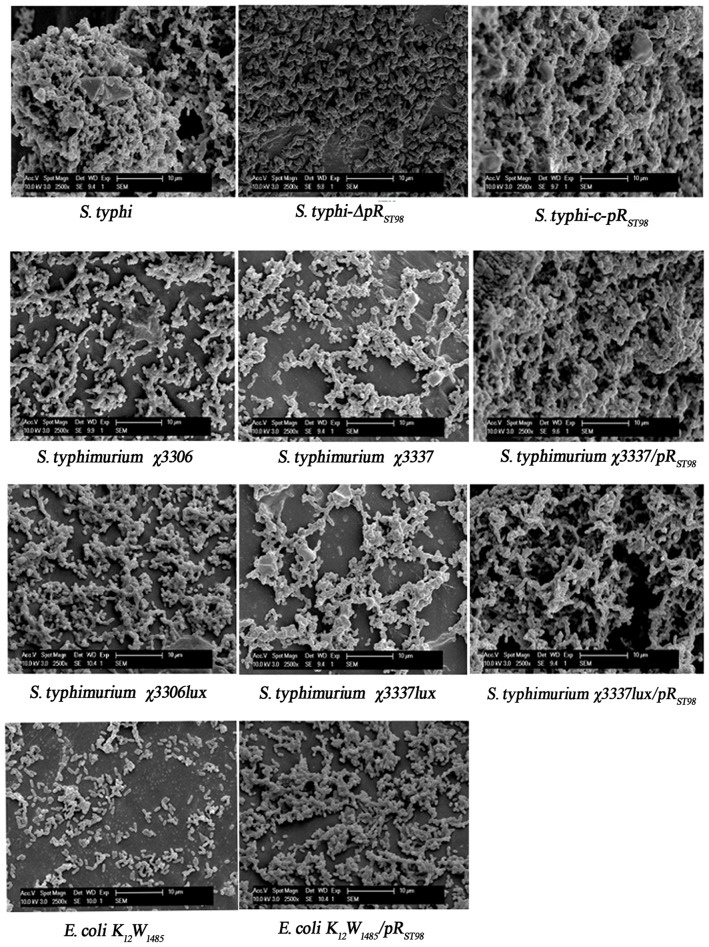
Observation of BF by SEM. Different bacteria were cultured in 24-well plates for 3 d, and the developed pellicles were harvested, placed on glass slides, and subjected to SEM.

SEM provides a detailed view of the connections in a bacterial community. Bacteria harboring pR_ST98_ significantly promoted BF formation as indicated by SEM, which showed that bacteria forming three-dimensional BF structures were embedded within denser matrices. However, the BFs of bacteria that did not harbor pR_ST98_ were discontinuous and discretely patchy (**Fig. 4**). These results corroborate those from violet staining and CLSM, suggesting that pR_ST98_ promotes BF formation in all of the tested bacteria, including *S. typhi*, *S. Typhimurium*, and *E. coli*.

### 2. pR_ST98_ promotes BF formation in different bacteria *in vivo*

**Figure 5:**
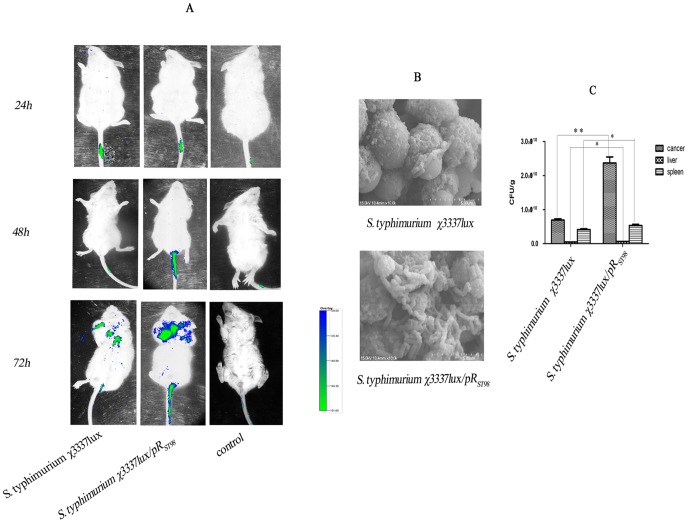
Bacterial accumulation at the indicated time points and the bacterial load in the organs of CT26 tumor mice. (**A**) Tumor-bearing mice were infected with 1×10^7^ CFU of *S. Typhimurium* χ3337*lux* and χ3337*lux*/pR_ST98_. The bioluminescence signals were captured by IVIS at the indicated time points. (**B**) Comparison of χ3337*lux* and χ3337*lux*/pR_ST98_ accumulated in tumors at 3 d p.i. by SEM. (**C**) CFU counts of tumors, livers and spleens infected by χ3337*lux* or χ3337*lux*/pR_ST98_. (**p <0.01); (*p <0.05)

To study the effect of the pR_ST98_ plasmid on bacterial proliferation and BF formation *in vivo*, we established two animal models, a tumor-bearing mouse model and a mouse urethral catheter model. Electrotransforming the bacteria with the *lux* gene made it possible to detect dissemination in tumor-bearing mice by a non-invasive method, and *lux* was shown to have no effect on bacterial growth. After intravenously infecting mice, *S. Typhimurium* quickly circulated within the blood in the bodies of the mice. It was found that χ3337*lux* and χ3337*lux*/pR_ST98_ accumulated preferentially in tumors detected by IVIS at 3 d p.i., and χ3337*lux*/pR_ST98_ in tumor emitted stronger bioluminescence signals than χ3337*lux* did, indicating that χ3337*lux*/pR_ST98_ formed thicker BFs. The same load of χ3337*lux*/pR_ST98_ was used to infect normal mice as a control, but no signal was observed at the desired sites (**Fig. 5A**), most likely due to the quick dissemination in the blood that was beyond the detection limit of IVIS. To further analyze the histological changes in infected mice and bacterial load, the tumor, livers and spleens were sterilely recovered based on the IVIS images at 3 d p.i. for SEM and CFU counting. Metastasis in livers and spleens by tumor cells, along with swelling organs, were found. The inflammation was more severe in the χ3337*lux*/pR_ST98_-infected group. Consistent with the results from IVIS, SEM showed that more χ3337*lux*/pR_ST98_ was accumulated in tumor. The livers and spleens from mice infected with χ3337*lux*/pR_ST98_ were loaded with more bacteria as well, indicating that the pR_ST98_ plasmid promoted bacterial spread and proliferation as well as enhancing virulence (**Fig. 5B and C**).

**Figure 6:**
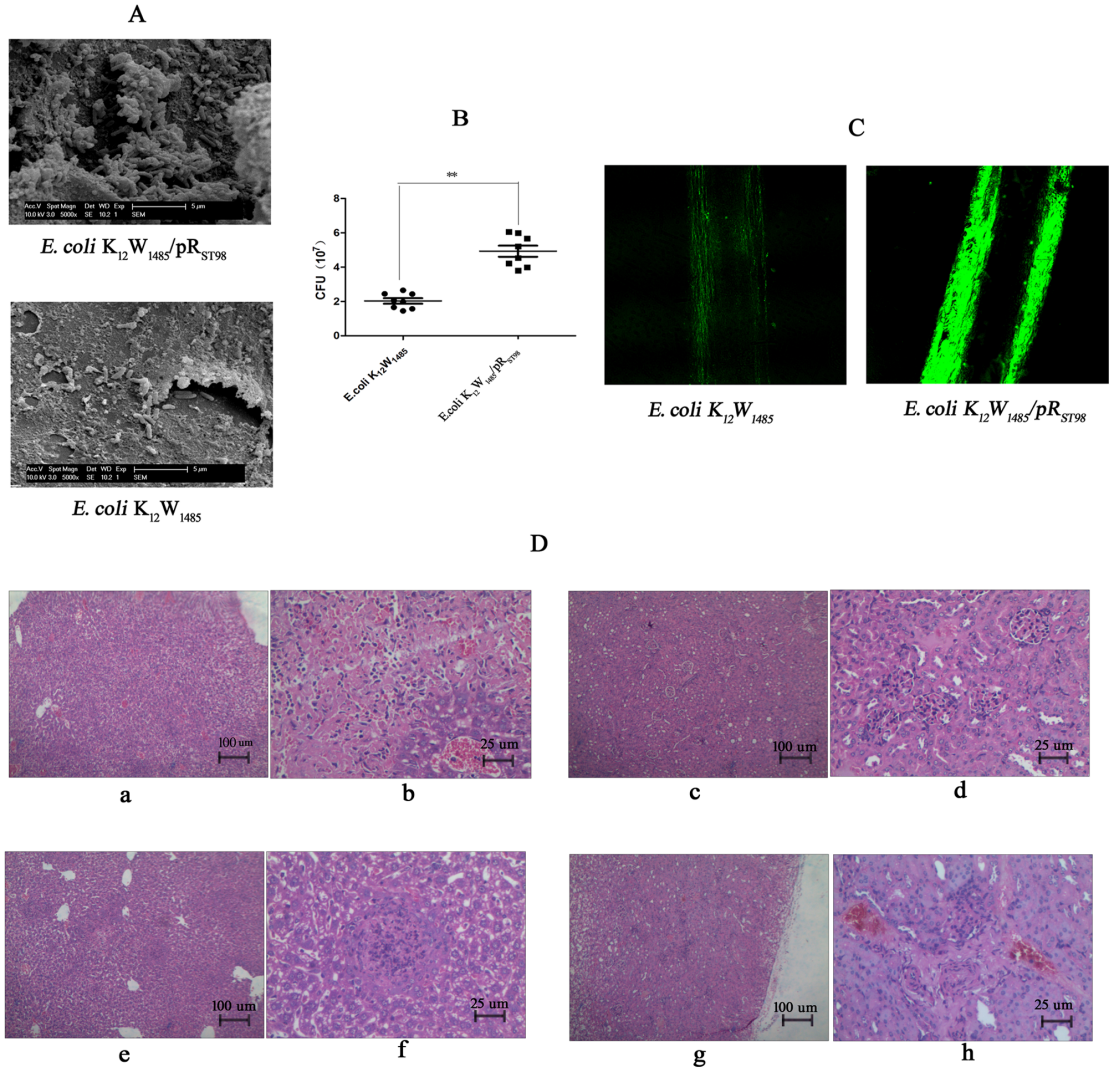
PE10 tubes recovered from the mouse urethral catheter model and histological changes of livers and kidneys. (**A**) Analysis of K_12_W_1485_ and K_12_W_1485_/pR_ST98_ BF on PE10 tubes at 5 d p.i. by SEM. (**B**) Quantification of K_12_W_1485_ and K_12_W_1485_/pR_ST98_ colonizing on PE10 tubes at 5 d p.i. (*p* <0.05). **Dots and dashes indicate the cfu of K_12_W_1485_ and K_12_W_1485_/pR_ST98_, respectively, recovered from BF on PE10 tubes. The middle long horizontal line represents the mean cfu, and the short line represents the SD**, (**p <0.01). (**C**) The tubes recovered from mice after urethral catheter at 5 d p.i. were washed with PBS and stained with AO, and bacteria were detected by CLSM. (**D**) H&E staining of livers and kidneys at 8 d after application of urethral catheter. (**a and b**), Livers of mice infected with K_12_W_1485_. (**c and d**), Kidneys of mice infected with K_12_W_1485_. (**e and f**), Livers of mice infected with K_12_W_1485_/R_ST98_. (**g and h**), Kidneys of mice infected with K_12_W_1485_/pR_ST98_.

PE10 tubes pre-incubated with *E. coli* were inserted into the mouse urethras. The mice were still active at 5 d post-insertion. Stable BFs of *E. coli* K_12_W_1485_/pR_ST98_ or *E. coli* K_12_W_1485_ developed on the surface of PE10 tubes were detected by CLSM after 5 d post-insertion under bright light. SEM, CLSM and CFU counting showed that the BFs formed by *E. coli* K_12_W_1485_/pR_ST98_ were thicker and had denser extracellular matrices compared with those in the control strain *E. coli* K_12_W_1485_ (*p* <0.05) (**Fig. 6A to C**). However, the livers and kidneys recovered from mice showed no pathological changes at 5 d post-insertion. When the insertion was extended to 8 d, sluggish behavior appeared in all mice, and more severe symptoms were observed in the *E. coli* K_12_W_1485_/pR_ST98_ group demonstrated by abdominal dropsy, swelling in livers and kidneys, and punctate lesions. The symptoms induced by *E. coli* K_12_W_1485_/pR_ST98_ BFs showed further histological changes in livers and kidneys by H&E staining, including inflammatory cell infiltration and severe damage in the hepatic lobule and the glomerular structure (**Fig. 6D**). At 12 d post-insertion, most of the mice infected with *E. coli* K_12_W_1485_/pR_ST98_ died, while the mice with *E. coli* K_12_W_1485_ infection survived longer than 17 d after insertion.

### 3. C8-AHLs enhances bacterial adherence, resistance, *rck* locus and transcription and bacterial BF formation

**Figure 7:**
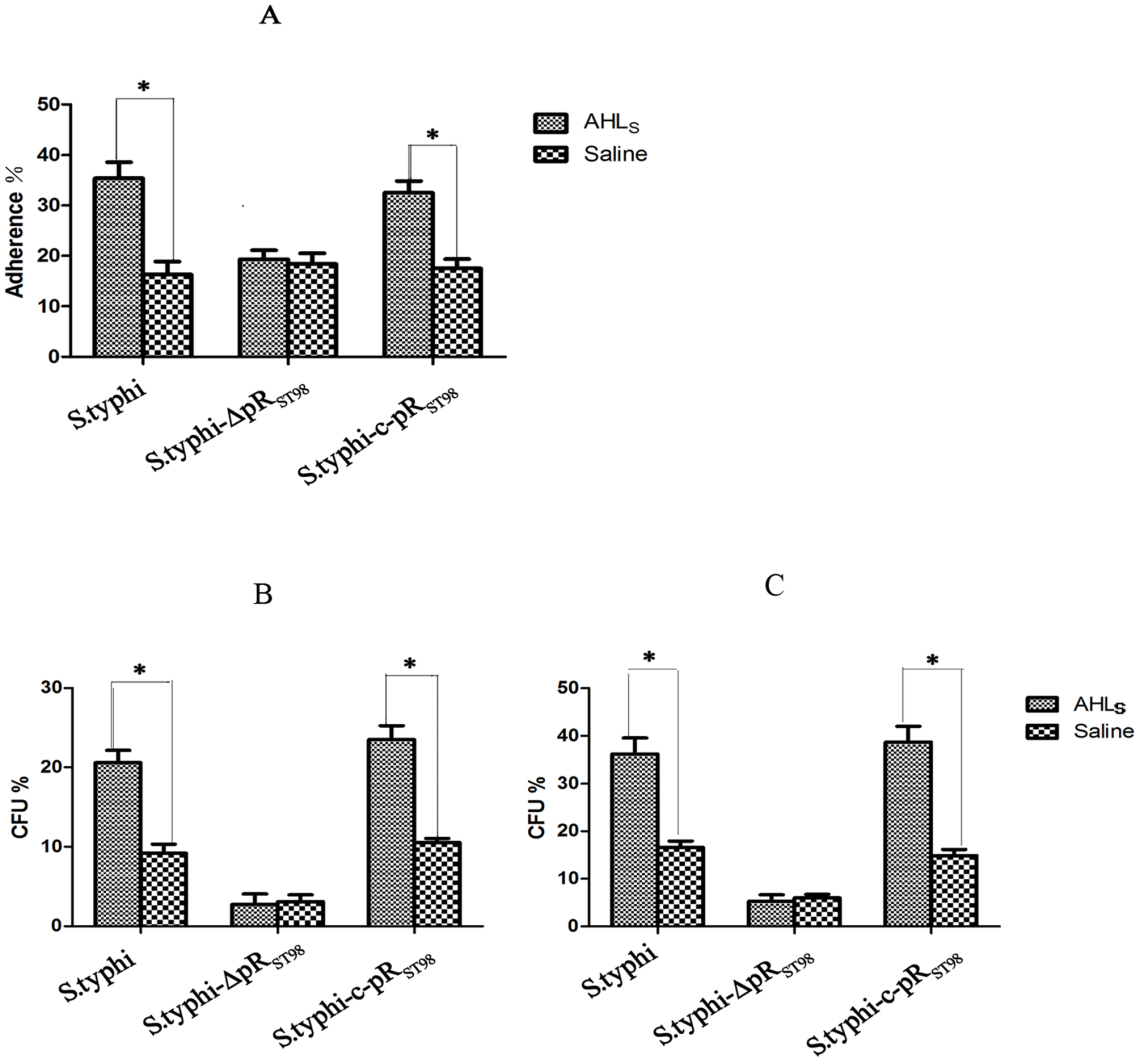
The effect of AHLs on *rck* expression and its related function. (A) The adherence rate of *S. Typhi* to HeLa cells in the presence of AHLs (*p <0.05). (B and C) Quantification by CFU of surviving bacteria after incubation with sera from rabbits (B) and guinea pigs (C) in the presence of AHLs and saline (*p <0.05).

AHLs, signaling molecule of the QS system, were shown to effect the BF formation in *E. coli* and the bacterial adherence [16]. To determine whether AHLs have similar effects on the BF formation in *Salmonella*, bacterial adherence assays were performed in the ST_8_ group treated with C8-AHLs. It was found that ST_8_ and ST_8_-c-pR_ST98_ displayed higher adherence rate than ST_8_-ΔpR_ST98_ (*p* <0.05), while no difference was observed for the adherence rate between ST_8_ and ST_8_-c-pR_ST98_ (*p*> 0.05). As for the control group (treated with saline), the adherence of the three strains to HeLa cells was similar (*p*> 0.05). ST_8_ and ST_8_-c-pR_ST98_ incubated with C8-AHLs showed more adherence than with saline (**Fig. 7A**). This result indicates that AHLs promoted bacterial adherence, on which pR_ST98_ may have an effect.

AHLs promote BF formation in *E. coli*, which subsequently increases bacterial resistance against hostile factors including serum. To investigate whether AHLs enhanced *Salmonella* resistance, a complement-mediated killing assay was performed. When incubating with rabbit and guinea pig serum, the survival rate of ST_8_ and ST_8_-c-pR_ST98_ treated with C8-AHLs significantly increased compared to the survival in the control group treated with saline, suggesting that AHLs enhanced *Salmonella* resistance. Furthermore, pR_ST98_ was indicated to participate in this process because ST_8_ and ST_8_-c-pR_ST98_ showed more resistance against killing by serum than ST_8_-ΔpR_ST98_ (*p* <0.05). Meanwhile, no significant difference was observed between ST_8_-ΔpR_ST98_ treated with or without C8-AHLs for their survival in serum (*p*> 0.05) (**Figs 7B and C**).

**Figure 8:**
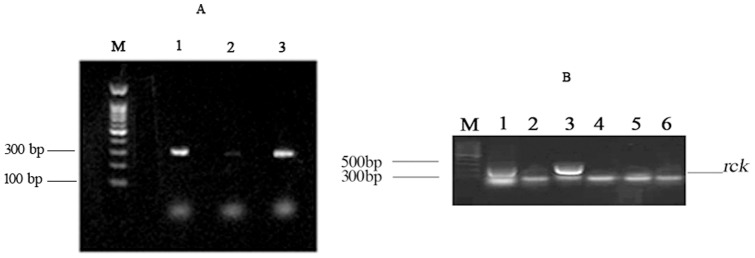
The locus of *rck* and its expression. (A) PCR of *rck* gene in pR_ST98_. M: 1000 bp DNA ladder; Lane 1: *S. Typhi* ST_8_; Lane 2: *S. Typhi* ST_8_-ΔpR_ST98_; Lane 3: *S. Typhi* ST_8_-c-pR_ST98_. (B) The effect of AHLs on the expression of the *rck* gene. M: 1000 bp DNA ladder; Lane 1: *S. Typhi* ST_8_ treated with AHLs; Lane 2: *S. Typhi* ST_8_ treated with saline; Lane 3: *S. typhi* ST_8_-c-pR_ST98_ treated with AHLs; Lane 4: *S. Typhi* ST_8_-c-pR_ST98_ treated with saline; Lane 5: *S. Typhi* ST_8_-ΔpR_ST98_ treated with AHLs; Lane 6: *S. Typhi* ST_8_-ΔpR_ST98_ treated with saline.

It was reported that *rck* located on the virulence plasmid of some serotypes of *Salmonella*, whose expression is regulated by the AHL receptor, effects the expression of plasmid-encoded fimbriae. In this study, it was proven that the *rck* gene was located on pR_ST98_ (**Fig. 8A**). To measure *rck* expression in the presence of AHLs and its relationship with pR_ST98_, the transcription of *rck* was measured in the ST_8_ group treated with C8-AHLs or saline. RT-PCR results showed that *rck* was only expressed in ST_8_ and ST_8_-c-pR_ST98_ strains treated C8-AHLs but not in strains treated with saline. *rck* was not detected *in* ST_8_-ΔpR_ST98_ treated with C8-AHLs or saline (**Fig. 8B**). These results indicated that the expression of *rck* was stimulated by C8-AHLs.

**Figure 9:**
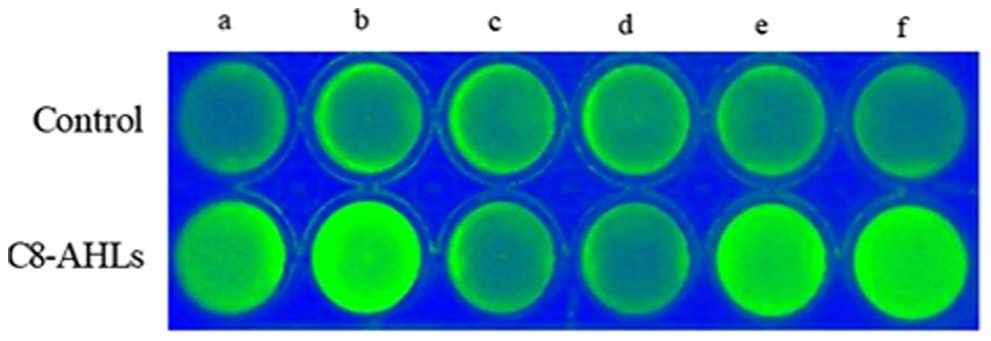
AHLs on *S. Typhi* BF formation. *S. Typhi*, cultured in 24-well polystyrene plates for 24 h by adding 1µM C8-AHLs and 1µM saline and detected by IVIS. a,b: ST_8_ *lux*; c,d: ST_8_-ΔpR_ST98_ *lux*; e,f: ST_8_-c-pR_ST98_ *lux*.

To further determine whether AHLs have effects on BF formation in *Salmonella*, BF formation assays were performed in the ST_8_ *lux* group treated with C8-AHLs. Compared with the control group, C8-AHLs significantly enhanced bacterial BF formation. In the C8-AHLs group, ST_8_ *lux* and ST_8_-c-pR_ST98_ *lux* emitted brighter fluorescence signals than ST_8_-ΔpR_ST98_ *lux* did, indicating that C8-AHLs promoted BF formation in ST_8_ and ST_8_-c-pR_ST98_ (**Fig. 9**).

## Discussion

In response to limited nutrients and stressful conditions, many microorganisms form BFs by secreting polymeric matrices to interweave individual cells and build structural communities on abiotic or living surfaces. Due to the significance of BF formation in increasing the resistance of bacteria against hostile environments, BFs have become a significant research interest in the medical, food and environmental fields.

Jean-Marc Ghigo first found that natural conjugative plasmids have the capability of promoting BF formation in *E. Coli* [15]. In addition, bacteria harboring conjugative plasmids developed thicker BFs than those not harboring such plasmids. However, the relationship between the conjugative plasmids in *Salmonella* and BF formation has not been studied.

The effects of pR_ST98_ on BF formation were explored in this study. Crystal violet staining, SEM and CLSM revealed that *S. Typhi*, *S. Typhimurium* and *E. coli* harboring pR_ST98_ formed thicker BF *in vitro*, compared with the isogenic strains not carrying pR_ST98_. It was also observed that *S. Typhimurium* χ3306 and χ3337 had similar abilities to form BFs, which is inconsistent with the study of Teodósio JS *et al* [29]. We speculated the different plasmids and BF-producing systems may contribute to this inconsistency. We noticed that *E. coli* K_12_W_1485_/pR_ST98_ had a weak ability to form BFs compared with *Salmonella* strains harboring pR_ST98_. This heterogeneity in BF formation may arise because the synthesis of extracellular polymeric substances (EPS) in *Salmonella* outcompetes that in *E. coli* in medium, as reported by Rong Wang *et al.* Regarding the heterogeneity in the promotion of BFs by conjugative plasmids, Røder HL *et al.* proposed that the different genetic backgrounds of the plasmid-harboring hosts may account for different BF formation when the same plasmid was used [30]. Our previous study demonstrated that in different genera, the conjugal transfer conditions of the pR_ST98_ plasmid were different *in vitro* or in mice, and the resistance markers encoded by the same plasmid varied in different strains, which showed the diversity and complexity of the gene expression from the plasmid. Thus, the effects of BF formation by different plasmids in various hosts may demand specific analysis.

In animal experiments, a tumor bearing mouse model was used to study the effects of pR_ST98_ on BF formation in *S. Typhimurium*, which was used as a surrogate of *S. Typhi* because no animal model is available for *S. Typhi* infection. In the tumor-bearing mouse model, χ3337*lux*/pR_ST98_ was found preferentially in tumors with a considerably larger amount than χ3337*lux*. The observation that solid tumors are treatable via bacterial infection was made previously [31]–[32]. Colonization of bacteria on solid tumors could cause growth retardation or even the complete elimination of the tumors [33]. pR_ST98_ promoting host bacterial BF formation may have a therapeutic potential in fighting against tumors. Furthermore, our invasion study *in vitro* proved that bacteria in BFs showed a lower invasion ability compared with the corresponding planktonic form (data not shown), which is consistent with the finding by Katja Crull *et al.* that BF-forming bacteria did not invade intracellularly *in vivo* after they established BFs. The intracellular invasion by *Salmonella* may be due to the differential expression of invasive genes on *Salmonella* pathogenicity island 1 (SPI-1) induced by BF formation [34].

Another animal model, a mouse urethral catheter model, was established to study the effects of pR_ST98_ in *E. coli* on BF formation *in vivo*. *E. coli* K_12_W_1485_/pR_ST98_ was found to form only discrete patchy BFs at 3 d post-implantation, while *E. coli* K_12_W_1485_ was not detected in tubes until 5 d post-implantation (data not shown). *E. coli* K_12_W_1485_/pR_ST98_ developed denser BFs at 5 d post-implantation, in line with bacterial titers recovered from established BFs on tubes. No histological changes were observed in the livers and kidneys of either group. When the implantation with tubes pre-incubated with *E. coli* was extended to 8 d or beyond, more severe inflammation was observed. Significantly, *S. Typhimurium* χ3337*lux*/pR_ST98_ caused more severe inflammation in organs than χ3337*lux* did. A similar phenomenon was observed for *E. coli* K_12_W_1485_/pR_ST98_ and K_12_W_1485_. These results indicate that pR_ST98_ aggravates the infection by promoting BF formation. Recently Rong Wang and Victoria J. Savage *et al*. demonstrated that the BF increases horizontal transfer of multi-resistant conjugative plasmids to plasmid-free bacteria compared to planktonic bacteria [35]–[36]. Therefore, it seems that conjugative plasmids facilitate BF formation, and vice versa. Therefore, given the intestinal origin and the conjugative transfer of pR_ST98_, interaction between pR_ST98_ and BF may make *Salmonella* infections worsen.

QS, a bacterial communication system, has been implicated in BF formation. To date, three types of *Salmonella*-associated QS signals have been described as AHLs, autoinducer-2 (AI-2) and autoinducer-3 (AI-3). However, the study on AI-2 and AI-3 revealed their minor roles in *Salmonella* BF formation in some conditions. While *Salmonella* does not produce AHLs, the AHL receptor SdiA was found in *Salmonella* to sense exogenous AHL signals to influence BF formation. A recent study revealed that the presence of SdiA enhances *E. coli* O157:H7 (O157) colonization and persistence in fecal shedding of the bovine large intestine, the prerequisites for developing a BF. Rck is a 17-kDa outer-membrane protein encoded by the *rck* gene located on the virulence plasmid of *Salmonella enterica* serovars Enteritidis and Typhimurium. The expression of *rck* in both *E. coli* and *S. Typhimurium* confers bacterial resistance against complement-mediated killing [37]. Rck is homologous to *Yersinia enterocolitica* Ail, which is capable of influencing bacterial adherence to epithelial cell lines [38]. We hypothesized that *rck* may influence BF formation. In the present study, it was proven that the *rck* gene was located on pR_ST98_, and *rck*-containing pR_ST98_ and C8-AHLs enhanced the cellular adherence of bacteria harboring pR_ST98_ and increased bacterial resistance against serum by activating transcription of *rck*. In addition, C8-AHLs promoted BF formation in bacteria containing pR_ST98_. These results partially explained the pR_ST98_-mediated BF promotion.

The mechanism of the effects of conjugative plasmids on BF formation is certainly complex and reciprocal. It is not clear whether the reported explanations could be applied to this study, although the studies on the mechanism may provide some clues. Further investigations will be focused on the factors that contribute to pR_ST98_-mediated BF formation and the mechanisms associated with the heterogeneity in BF formation.

Taken together, we demonstrated that the conjugative plasmid pR_ST98_, which was isolated from *S. typhi*, can promote BF formation in intestinal bacteria such as *S. Typhi*, *S. Typhimurium*, and *E. coli*. Animal models showed that pR_ST98_ promotes BF formation in *S. Typhimurium* and *E. coli*. In attempting to investigate the underlying mechanism, we found that the transcription of *rck* located on pR_ST98_ is activated by C8-AHLs. Therefore, it is reasonable to conclude that pR_ST98_ promotes BF formation in its host bacteria through the AHLs-SdiA-Rck pathway. The relationship between the conjugative plasmid pR_ST98_ and BF formation could provide insights into the prevention and treatment of *Salmonella* BF-related disease and intestinal infection.
